# Data for improvement and clinical excellence: protocol for an audit with feedback intervention in home care and supportive living

**DOI:** 10.1186/1748-5908-7-4

**Published:** 2012-01-18

**Authors:** Kimberly D Fraser, Anne E Sales, Hannah M O'Rourke, Corinne Schalm

**Affiliations:** 1 Faculty of Nursing, University of Alberta, Edmonton, Alberta, Canada; 2 VA Inpatient Evaluation Center, Ann Arbor VA HSR&D Center of Excellence, Ann Arbor, MI, USA; 3 Faculty of Nursing,, University of Alberta, Edmonton, Alberta, Canada; 4 Shepherd's Care Foundation, Edmonton, Alberta, Canada

## Abstract

**Background:**

Although considerable evidence exists about the effectiveness of audit coupled with feedback, very few audit-with-feedback interventions have been done in either home care or supportive living settings to date. With little history of audit and feedback in home care or supportive living there is potential for greater effects, at least initially. This study extends the work of an earlier study designed to assess the effects of an audit-with-feedback intervention. It will be delivered quarterly over a one-year period in seven home care offices and 11 supportive living sites. The research questions are the same as in the first study but in a different environment. They are as follows:

1. What effects do feedback reports have on processes and outcomes over time?

2. How do different provider groups in home care and supportive living sites respond to feedback reports based on quality indicator data?

**Methods:**

The research team conducting this study includes researchers and decision makers in continuing care in the province of Alberta, Canada. The intervention consists of quarterly feedback reports in 19 home care offices and supportive living sites across Alberta. Data for the feedback reports are based on the Resident Assessment Instrument Home Care tool, a standardized instrument mandated for use in home care and supportive living environments throughout Alberta. The feedback reports consist of one page, printed front and back, presenting both graphic and textual information. Reports are delivered to all employees working in each site. The primary evaluation uses a controlled interrupted time-series design, both adjusted and unadjusted for covariates. The concurrent process evaluation includes observation, focus groups, and self-reports to assess uptake of the feedback reports. The project described in this protocol follows a similar intervention conducted in our previous study, Data for Improvement and Clinical Excellence--Long-Term Care. We will offer dissemination strategies and spread of the feedback report approach in several ways suited to various audiences and stakeholders throughout Alberta.

**Significance:**

This study will generate knowledge about the effects of an audit with feedback intervention in home care and supportive living settings. Our dissemination activities will focus on supporting sites to continue to use the Resident Assessment Instrument data in their quality improvement activities.

## Background

Although there is evidence about interventions to improve quality of care in some healthcare settings, the evidence for quality-improvement interventions in long-term care (LTC) is variable [1-7]. Specific to audit and feedback interventions, there is little evidence on utility of this type of intervention in continuing care settings. With unprecedented growth in both home care (HC) and supportive living (SL), data about the quality of care as well as about how to improve quality of care in these settings are a priority. Subsequently, we did a study on an audit-with-feedback intervention across the three streams of continuing care: LTC, HC, and SL. The protocol we report in this paper is on the second phase of our study that will be carried out in HC and SL. The study protocol for the first phase in LTC was previously reported [1]. In this second phase, we expand the original protocol to cover HC and SL settings.

The number of healthcare resources needed to meet the needs of the large and ever-growing HC and SL sector populations is a top priority of healthcare organizations today. HC is defined as an array of services designed to meet individual and family needs [8,9]. SL is continuing care, usually in congregate living settings, and is also referred to as assisted living or designated assisted living in some sectors. The services vary from setting to setting to a degree but are primarily home care and support services to seniors. The Canadian Home Care Association reported that from 1995 to 2006, the number of publicly funded HC clients grew by nearly 100% to approximately one million clients at any given time [9-12]. Further, the Health Council of Canada reported that from 1995 to 2002, the number of Canadians receiving publicly funded HC increased by 60%. The need to deliver quality care to this growing population is essential and must be done in an efficient and effective manner in order to maximize our limited healthcare resources [9,11]. In the Government of Alberta's strategy, Aging in the Right Place, various initiatives are outlined to address the increasing healthcare needs of HC and SL clients [13]. The strategies contained within Aging in the Right Place involve enhancing HC and SL through improved assessment, expansion of current programs, introduction of appropriate emergency supports, and facilitation of transitions from acute care back into the community [13]. One outcome is that people receive the right care at the right time by the right provider. These actions are further supported in the Alberta Continuing Care Association document on implementing recommendations of the Ministry Advisory Committee on Health (MACH) [14,15]. Within the MACH report, there is an emphasis on incorporating the pillars of acceptability, accessibility, appropriateness, effectiveness, efficiency, and safety through the use of evidence-based research into SL facilities [14]. In order to mobilize an effective healthcare workforce, it is essential that evidence-based practice be at the forefront to ensure a high quality of care for HC and SL clients. In order to adequately attain a relevant and meaningful level of knowledge among community healthcare providers, HC and SL client assessment data must be incorporated into research-based knowledge-translation (KT) strategies [16]. We position quality-improvement initiatives, such as audit and feedback, as a KT strategy.

Despite the aforementioned literature, there is a dearth of evidence from HC and SL settings as these sectors have received relatively little attention in terms of implementation of evidence-based practice, or at least little that is reported in the literature. Further, very little research has been done to examine the utility of client assessment data on improving nursing care in the HC and SL sectors. The Resident Assessment Instrument-Home Care (RAI-HC) tool belongs to a suite of tools used by organizations to collect, analyze, and understand the health status of clients. The RAI tools help to standardize client assessment and form an evidence base to influence clinical practice and policy decisions and thus have been mandated by many organizations [17]. Within community settings, such as HC and SL, the RAI-HC is a useful tool for highlighting themes related to functioning and quality of life of clients [18]. The RAI-HC quality indicators can be assessed and recorded and include categories such as pain, falls, or depression [18]. The assessment data contained within the RAI-HC is easily accessible and, therefore, can be used to assess trends or patterns in client quality indicators.

The detection of patterns and trends within a particular RAI-HC data set can be further utilized to describe areas in which quality-of-care improvements could be made through increased use of evidence-based practice. In order to increase use of evidence-based practice and subsequently improve care in HC and SL, the relationships between the assessment findings and their usefulness and applicability, including how well they are understood by healthcare providers, must be explored; one way to do this is through the use of audit and feedback as a quality-improvement strategy.

### Primary purpose and study objectives

The primary purpose of the Data for Improvement and Clinical Excellence (DICE) project is to assess the effects of an audit with feedback intervention in all three streams of continuing care: LTC, HC, and SL. The LTC intervention has been recently completed and is in the monitoring phase [1]. The HC and SL audit-with-feedback intervention will be delivered quarterly over a one-year period in seven HC offices and 11 SL sites across Alberta, using data from the RAI-HC.

We address these research questions:

1. What effects do RAI feedback reports have on processes and outcomes over time?

2. How do different provider groups in HC and SL respond to feedback reports based on RAI-HC quality indicator data?

## Methods

The overall intervention evaluation uses a controlled interrupted time-series design with quarterly feedback reports in the seven HC offices and 11 SL sites across Alberta. Follow-up surveys to assess uptake of the audit-with-feedback intervention will be offered to all participating employees one week after feedback report distribution. The purpose of the follow-up survey is to assess staff response to the feedback reports and their intent to change their behavior based on what they learned from the feedback. We will not be measuring actual change in behavior through the surveys but rather their *intent *to change their behavior. This is similar to the survey tool that was used in the Data for Improvement and Clinical Excellence--Long-Term Care (DICE-LTC) study, with only wording revised to suit the HC/SL environment. A sample survey instrument can be found in Additional File 1.

We will use in-person audit and feedback in two zones and electronic distribution in two zones. This will be more fully described later in this protocol paper.

The process evaluation will be conducted concurrently with the prospectively collected survey data and will consist of observation, focus groups, and self-report measures to assess uptake of the feedback reports. We define uptake as reading the feedback reports, discussing with colleagues and managers, and reporting some degree of intention to change behavior based on the reports. Observation will occur in the sites where we deliver the feedback reports in person. The focus groups will be targeted sessions based on the findings of the observations and the self-report survey and will be held in four of the participating sites.

We received ethics approval from the University of Alberta Health Research Ethics Board, panel B. We obtained operational approval for all sites from all participating study organizations. Participation by staff is voluntary and will not affect their employment in any way.

### Project team

The project team comprised both researchers and decision makers who had existing relationships prior to this project, primarily through the Knowledge Brokering Group (KBG). The KBG team was focused on using data to inform decision making in continuing care settings. The overall makeup of the team and each member's background is described in the DICE-LTC protocol paper [1]. The specific program funding for this project requires active collaboration between researchers and decision makers [19], and the team works on a linkage and exchange, integrated KT model. New team members were added for this second phase in HC and SL, and again, the researcher lead and decision-maker partners had existing collaborative relationships. We provide an updated description of the project team for DICE-HC in Additional File 2.

### Settings and sample

The settings are seven HC offices and 11 SL sites across Alberta, Canada. The selected HC sites are part of Alberta Health Services (AHS) and their direct care providers and managers are employees of AHS, whereas the SL sites are operated by provider organizations under contract to AHS and the direct care providers and managers are employees of the provider organization. However, in all sites, case management and assessments are carried out by case managers who are part of AHS. The sites have all implemented the RAI-HC instrument [20].

### Recruitment

We recruited HC offices and SL sites in rural and urban areas across four zones in Alberta (North, Central, Calgary, and South) to participate [21]. Ideally, by the time we begin feedback reports, clients/residents of these HC office and SL sites would have been assessed using RAI-HC at least once and preferably more than once. The degree of RAI-HC implementation guided our choice of sites, as most HC and SL sites in Alberta began implementation of RAI-HC in 2007. The roll-out was staggered across the province and was fully implemented by July 2011 and, going forward, all new client assessments and re-assessments will be completed using the RAI-HC. We recruited HC offices and SL sites with at least 10 nonregulated staff, as well as at least 20 to 30 clients/residents being assessed using the RAI-HC tool in order to obtain a critical mass. We recruited direct care employees from all of the professions, disciplines, and groups in the participating sites. Participants may include site managers, case managers, registered nurses, licensed practical nurses, healthcare aides, occupational therapists and assistants, recreational therapists and assistants, physical therapists and assistants, pharmacists, and social workers.

### The intervention

#### Procedures for feedback report generation and distribution

Feedback report distribution and data collection will occur in two ways: either in person or electronically. This was done for two reasons: first, logistically given the geographic size of Alberta and the desire to include most zones and, second, to assess the difference in response and utility between in-person and electronic feedback reports. We will use the Alberta Context Tool (ACT) to assess context in the sites as we did in DICE-LTC [1,21,22]. The ACT will be administered at the beginning of the project immediately following in-person information sessions and prior to beginning report distribution. Information sessions and administration of the ACT tool during the information sessions will occur in-person for all sites across the province.

Unlike previous studies using feedback for quality improvement, this study focuses on the individual rather than the whole organization [23], and front-line staff are targeted to receive the feedback reports directly. Thus, our purpose is to provide the feedback reports, generated using RAI-HC data, directly to front-line staff using either an in-person distribution approach (North and Central zones) or via electronic distribution (Calgary and South zones). The RAI-HC covers a wide range of process and outcome data at the individual client level, and assessments are updated annually for each client unless there is a change in a client's health status or their functional or cognitive status, or if the client has been admitted to hospital. The quality indicators we have chosen to include in the feedback report are negative mood, risk of falls, pain, delirium, and visits to the hospital in the last 90 days. These five areas are the top domains identified as important by HC and SL staff. They were selected in 2010 using a voting process completed by AHS senior leadership, who consulted with their managers and/or direct care employees. The RAI-HC data will also be used to measure client-level outcomes.

Data will be extracted by AHS for each of the participating sites. All personal identifiers will be removed prior to extraction at AHS and delivery to the researchers. The data extraction for each quarter will include all assessments completed within the prior 12-month period so that in each quarterly report we will capture one year's worth of assessment data. The anonymized data will be submitted to the research team who will generate feedback reports.

The feedback reports are full color line graphs with minimal text on one double-sided page. An example of a feedback report is provided in Additional File 3. The reports will be distributed quarterly for one year. The first and third quarterly reports will include pain, negative mood, and falls. The second and last quarterly reports will also include delirium and visits to the hospital in the last 90 days. Each report will have the site's own data, with aggregate data from all other sites to compare their performance on the specific quality indicators to all other participating sites.

We will distribute feedback reports to all available employees at the participating sites once each quarter. We elected to do quarterly feedback reports rather than monthly feedback reports for two reasons. First, it is unlikely that there will be enough new or repeat assessments completed each month to yield enough data for reliable estimates. Second, based on our observations in DICE-LTC, we did not want to burden participants with monthly reports.

The following is a description of the in-person distribution approach, followed by the modifications that we will make for the sites that are participating electronically. All of our visits will be prearranged with the site champions and during a time where we are most likely to capture the most staff on-site, for example, immediately following a team meeting when all or most staff come together at the SL site or HC office. In SL sites, there is often a huge campus, or several small sites, so it is not practical to try to reach staff outside of prearranged times. It is a similar case in HC, where staff are not in a central location, except during team meetings or in-services.

Once every three months (*i.e*., every quarter), two research assistants (RAs) will hand deliver the report to all employees present and available at each site during the time he or she is there. The reports are specific to the HC or SL site, and all direct care providers and managers within these sites are targeted to receive the report that gives them information about how the clients in their site compare to the other sites in the study on the selected indicator areas. Additional copies will be left in a central location, and staff will be told that they are welcome to take one later if they would like. The RAs will stay for about one hour after distributing reports to answer any questions or take comments. One of the RAs will also passively observe what employees do with the reports after receiving them and will discretely note how many times the employees read the reports, discuss them with others, discuss with the RA, or other actions such as throwing the reports away. No names or identifying information will be noted. We will use a simple check sheet as well as write narrative notes about the specific observations. This is the basis for our field notes and will be used in conjunction with staff self-report on the post-feedback survey to obtain an estimate of report uptake. At the end of the site visit, we will also post flyers reminding staff that we will be conducting surveys the following week.

##### Process evaluation

The purpose of the process evaluation is to assess both feedback report uptake and intention to change behavior. This component of our evaluation will assess whether staff read and understood the report and whether they intend to change a particular behavior that was targeted on the feedback reports. As in DICE-LTC, about a week after we distribute the feedback reports, we will ask employees to complete a survey to assess their response to the feedback reports. Staff will self-report on whether they received the report, whether they read the report, how they might use the feedback reports, whether they intend to use the information from the report to change care practices, and what sorts of changes they might consider making to their care practices as a result of the reports. All of these questions relate to feedback report uptake. The final section of the survey was constructed to capture intention to change behavior in pain assessment practice, according to the constructs described in a manual on survey development using the Theory of Planned Behavior [24,25]. This section will be completed by the direct care provider staff only. Surveys will be conducted in a room, away from client/resident care and activity. All surveys will be anonymous. We will ask some demographic information, including the office/site they work in and their years of experience in HC/SL settings. This is similar to the tool that was used in DICE -LTC, with only wording revised to suit the HC/SL environment. After completing the survey, each employee will also be offered a $5 Tim Horton's gift certificate in appreciation for their participation.

We will deliver the surveys to the sites during a time they are already meeting for another purpose. We will provide a self-addressed return envelope for returning the surveys to the research team. Our site champions are instrumental in the implementation of this study. Site champions will distribute the feedback report and survey to all employees who are not in attendance at the time of in-person report distribution using their usual mail distribution system. Those absent employees will review the feedback reports and complete the survey and return it to the research team in the self-addressed envelope provided.

In addition, we will conduct several 45-minute focus groups with regulated and unregulated healthcare providers at selected sites to further explore and verify the quantitative findings from the post-feedback surveys. The intention of the focus groups is to delve deeper into how the respondents felt they understood the reports and their perceptions of report utility. We will conduct the focus groups with 5 to 10 healthcare providers per group, which will take place after the report/survey distribution in the third quarter. The focus groups will use a semi-structured discussion format. We will derive the questions to stimulate discussion based on our preliminary survey data from the first two quarters, discussing in more depth several variables that were addressed in the quantitative analysis related to the perceptions of the feedback reports.

#### Procedures for electronic feedback report distribution

We will follow the same order as with North and Central zones described above, but rather than in person, we will distribute feedback reports and conduct the post-feedback report survey electronically. We will use the continuing care desktop (CCD), a tool developed by the Centre for Health Evidence in collaboration with Alberta Health and Wellness that is available to all employees in continuing care throughout the province of Alberta [26]. Access to the CCD is through individual log-in privileges managed through the workplace. We plan to post the feedback reports by site on the CCD, with a separate notice emailed to the site leader. The notice will include a poster that the site leader will be asked to print and post in at least three prominent locations to notify the employees to review the feedback report on the desktop. One week later, we will follow the same procedure with the site leader with a poster notifying the employees that the feedback report follow-up survey is available for them to complete. The participating employees will log onto the CCD, where they will see a link to complete the online survey. By using an electronic distribution mechanism, we will be able to compare, descriptively, how electronic feedback reports compare to in-person feedback reports. As well, comparisons on distribution for post-feedback report surveys will also be made between in-person and electronic formats.

### Analysis

Our primary analysis, using time series with and without adjustment for covariates, including site-level context data from the ACT survey, will allow us to assess change in the client outcomes that are reported in the feedback report over time. For the primary analysis for this study, we will pool the RAI-HC data from all study sites, using RAI-HC data for six months prior to the intervention, during the intervention, and for six months after the intervention. We will assess changes in trends seen before, during, and after the intervention period for the client outcomes included in the feedback reports (pain, delirium, negative mood, falls, visits to hospital within previous 90 days) and other outcomes not included in the reports (*e.g*., pressure ulcers, incontinence, and social engagement). For the purpose of the time-series analysis, the quarterly data will be converted to monthly measures of aggregate client outcomes across all sites. Predictor variables will include the intervention dose, operationalized as the proportion of staff who were observed or who self-reported reading the reports. Data for control facilities will be requested and analyzed at the end of the post-surveillance period.

Qualitative data from the focus groups and from the narrative observation notes will be coded into themes, including specific barriers and facilitators to feedback report uptake. At the site level, these findings will be used alongside the post-feedback report survey data, the client outcome data, and the findings from the ACT tool to glean additional detail around contextual differences that may explain possible variations in responses to the feedback reports across sites.

### Timeline

The DICE-LTC was completed in February 2010 [1]. The second phase of DICE, implementing a feedback intervention in HC/SL settings using the RAI-HC, began April 2011.

**Figure 1:**
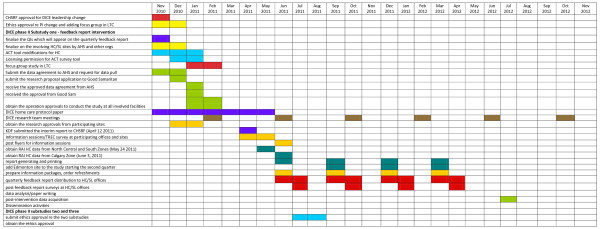
**This figure identifies the detailed project timeline for the DICE-HC study**.

Following a year-long intervention with quarterly report distribution to several HC offices/SL sites, DICE will enter its final year, focusing on dissemination and spread of the intervention throughout the province of Alberta. The full project timeline is provided in Figure 1.

### Dissemination and spread

The final phase of the study is focused on spread of approaches to using the RAI data to inform decision making in HC/SL contexts and will mirror dissemination activities used in the LTC phase [1]. In the final year of the DICE program, we will develop toolkits and training materials and will continue to work closely with the health region and managers within HC/SL settings that have expressed interest in continuing their engagement with a network of RAI users. After approaching zone leaders to obtain zone-level approval, we will contact administrators in HC/SL offices to request their voluntary participation. Our primary purpose is to offer the RAI coordinators in each of the settings tools, training, and technical support to implement their own local program of feedback report generation and distribution. This implementation effort will be evaluated using a one-time survey in each participating setting to assess response to the feedback reports from all staff. We will also request historical RAI-HC data to assess any change in outcomes from the year prior to the implementation to six months after the training. This monitoring period is shorter than ideal and will be extended if we can secure additional funding.

We believe this work to be relevant, timely, and occurring within a context where synergistic activities will support our dissemination plans. For example, *Putting RAI to Work: Network of RAI Data Users and Researchers *is a network of health authority representatives and organizations, funded through the Canadian Institutes for Health Research from 2008 to 2010, who have expressed interest in continuing their engagement in this network and in future work that focuses on using RAI data. They currently communicate their work with the RAI via an established website http://www.rairesun.ca/.

### Deliverables

The specific deliverables for this project will include (1) delineation of a process for identifying priorities across provider groups in continuing care settings in order to identify the core feedback report content, (2) a toolkit (manual and programming guides) for creating actionable feedback reports from the RAI data in HC/SL contexts, (3) maintenance of the website to connect the network of decision makers and researchers interested in using RAI data for developing and delivering feedback reports, and (4) a group of decision makers and researchers who have the skills and support to develop and use tools in HC/SL sites [1].

As previously reported, we believe our work will be an important contribution to both policy and practice in continuing care contexts beyond Alberta. In addition to providing important guidance about use of feedback reports in care settings, our highly structured approach may provide some guidance to researchers in implementation science in terms of organizing and planning audit-with-feedback interventions.

## Competing interests

AES is an Associate Editor of *Implementation Science*; all decisions on this paper were made by another editor. All other author(s) declare that they have no competing interests.

## Authors' contributions

AES conceived of the study, drafted and revised it, and is responsible for the conduct in LTC. CS conceived of the study, reviewed and contributed to drafts, and shares responsibility for its conduct. KDF conceptualized, drafted, and revised the second phase of DICE in HC and SL and is responsible for the conduct of this study in those streams of continuing care. HMO assisted in drafting and revising the second phase of DICE in HC and SL. All authors read and approved the final manuscript.

## Supplementary Material

Additional file 1 **Survey instrument**. This file contains an example of the post-feedback survey instrument. Click here for file

Additional file 2 **Study team**. This file contains the project team member descriptions at the time of implementation of DICE Phase II-Home care and Supportive Living. Click here for file

Additional file 3 **Feedback report**. This file contains an example of the feedback report used in Home care/Supportive living sites. Click here for file

## References

[B1] Sales AE Schalm C Data for improvement and clinical excellence: protocol for an audit with feedback intervention in long-term care Implement Sci 2010 5 74 1 7 20939926 10.1186/1748-5908-5-74 PMC2964554

[B2] Grimshaw J Eccles M Thomas R MacLennan G Ramsay C Fraser C Vale L Toward evidence-based quality improvement. Evidence (and its limitations) of the effectiveness of guideline dissemination and implementation strategies 1966-1998 J Gen Intern Med 2006 21 Suppl 2 S14 20 16637955 10.1111/j.1525-1497.2006.00357.x PMC2557130

[B3] Eccles M Walker A Walker A Johnston M Pitts N Changing the behaviour of healthcare professionals: the use of theory in promoting the uptake of research findings J Clin Epidemiol 2005 58 2 107 112 10.1016/j.jclinepi.2004.09.002 15680740

[B4] Foy R Eccles MP Jamtvedt G Young J Grimshaw JM Baker R What do we know about how to do audit and feedback? Pitfalls in applying evidence from a systematic review BMC Health Serv Res 2005 5 50 http://www.biomedcentral.com/1472-6963/5/50 10.1186/1472-6963-5-50 PMC1183206 16011811

[B5] Grimshaw J Eccles M Tetroe J Implementing clinical guidelines: current evidence and future implications J Contin Educ Health Prof 2004 24 Suppl 1 S31 7 15712775 10.1002/chp.1340240506

[B6] Jamtvedt G Young JM Kristoffersen DT O'Brien MA Oxman AD Audit and feedback: effects on professional pratice and health care outcomes Cochrane Database Syst Rev 2006 19 2 CD 000259 10.1002/14651858.CD000259.pub2 16625533

[B7] Shojania KG Grimshaw J Still no magic bullets: pursuing more rigorous research in quality improvement Am J Med 2004 116 11 778 780 10.1016/j.amjmed.2004.03.003 15144917

[B8] The Canadian Home Care Association Integrating and coordinating mental health and home care in Canada: a scan of promising practices and existing service delivery models 2008 http://www.cdnhomecare.ca/media.php?mid=1853

[B9] Fraser KD Estabrooks CA How do case managers make resource allocation decisions? A systematic review of the literature Med Decis Making 2008 28 394 410 10.1177/0272989X07312709 18480042

[B10] Government of Alberta Health and Wellness Supportive living framework 2007 http://www.continuingcare.gov.ab.ca.login.ezproxy.library.ualberta.ca

[B11] Fraser KD Estabrooks CA Allen M Strang VA Case manager resource allocation decision-making processes: A case illustration Care Manage J 2010 11 3 151 156 10.1891/1521-0987.11.3.151 20839480

[B12] Fraser KD Estabrooks CA Allen M Strang VA Factors that influence case managers' resource allocation decisions in pediatric home care: an ethnographic study IJNS 2009 46 337 349 10.1016/j.ijnurstu.2008.10.001 19019366

[B13] Government of Alberta Health and Wellness Continuing care strategy: aging in the right place 2008 http://www.health.alberta.ca.login.ezproxy.library.ualberta.ca/initiatives/continuing-care-strategy.html

[B14] Government of Alberta Health and Wellness Minister's advisory committee on health 2009 http://www.health.alberta.ca.login.ezproxy.library.ualberta.ca/initiatives/MACH-2009.html

[B15] Alberta Continuing Care Association Implementing the recommendations of the minister's advisory committee on health 2010 http://www.ab-cca.ca/news-and-articles

[B16] Tolson D Rolland Y Andrieu S Aquino J Beard J Benetos A Berrut G Coll-Planas L Dong B Forette F Franco A Franzoni S Sava A Swagerty D Trabucchi M Vellas B Volicer L Morley JE International association of gerontology and geriatrics: a global agenda for clinical research and quality of care in nursing homes J Am Med Dir Assoc 2011 12 3 184 189 10.1016/j.jamda.2010.12.013 21333919

[B17] Sales A O'Rourke H Draper K Teare GF Maxwell C Prioritizing information for quality improvement using resident assessment instrument data: experiences in one Canadian province Healthc Policy 2011 6 3 56 69 10.12927/hcpol.2011.22221 PMC3082388 22294992

[B18] Hawes C Fries BE James ML Guihan M Prospects and pitfalls: use of the RAI-HC assessment by the Department of Veterans Affairs for home care clients Gerontologist 2007 47 3 378 387 10.1093/geront/47.3.378 17565102

[B19] Canadian Health Services Research Foundation: Programs 2011 http://www.chsrf.ca.login.ezproxy.library.ualberta.ca/funding_opportunities/reiss/index_e.php

[B20] InterRAI: Instruments 2007 http://www.interrai.org/section/view/?fnode=10

[B21] Alberta Health Services AHS zone map 2011 http://www.albertahealthservices.ca/1532.asp

[B22] Estabrooks CA Squires JE Cummings GE Birdsell JM Norton PG Development and assessment of the Alberta Context Tool BMC Health Services Research 2009 9 234 [doi:10.1186/1472-6963-9-234] 10.1186/1472-6963-9-234 PMC2805628 20003531

[B23] Rantz MJ Popejoy L Petroski GF Madsen RW Mehr DR Zwygart-Stauffacher M Hicks LL Grando V Wipke-Tevis DD Bostick J Porter R Conn VS Maas M Randomized clinical trial of a quality improvement intervention in nursing homes Gerontologist 2001 41 4 525 538 10.1093/geront/41.4.525 11490051

[B24] Ajzen I The theory of planned behavior Organizational Behavior and Human Decision Processes 1991 50 179 211 10.1016/0749-5978(91)90020-T

[B25] Francis JJ Eccles M Johnston M Walker A Grimshaw J Foy R Kaner EFS Smith L Bonetti D Constructing questionnaires based on the theory of planned behaviour: A manual for health services researchers Center for Health Services Research 2004 University of Newcastle: Newcastle upon Tyne

[B26] Search Canada: Continuing care desktop 2006 http://www.searchca.net/users/FolderData/%7BD73864FB-FFCD-4BAE-B722-C91C6020D5B4%7D/search_continuing_care_flyer_2008_05.pdf

