# Supplementary material for: Data for improvement and clinical excellence: protocol for an audit with feedback intervention in home care and supportive living
Source: Implement Sci. 2012 Jan 18;7:4. doi: 10.1186/1748-5908-7-4 (PMC3292450; doi:10.1186/1748-5908-7-4)
Supplement: Additional file 1 — Survey instrument. This file contains an example of the post-feedback survey instrument. [file 1748-5908-7-4-S1.PDF]

Data for Improvement and Clinical Excellence (DICE)

Post-Feedback Report Survey:

General Information and Instructions:

BEFORE COMPLETING THIS SURVEY, please make sure you have received a copy of the feedback report.

This is an **anonymous** survey. None of the questions in this survey let us know who you are, and we are not asking you to identify yourself.

This survey has three sections:

Section A asks you some questions about yourself - which home care or supportive living site (called the 'site' in most of the survey) you work in, what your job title is, and how long you have been working in this area. We ask these questions so that we can compare answers among people who work in different jobs and in different sites.

Section B asks you about the DICE feedback report that was distributed in your site. We ask about how much of the report you read, understood, and how useful you felt it was. We also ask several questions to find out how you think you might use the information in the report. If you work in or cover more than one site in your usual work, please respond to the questions in this section based on the site report that you received.

Section C asks questions about whether you plan to change any of the ways you find out if a client is having pain, which only applies to people who give direct care to clients.

Note: We use the term "client" here but do recognize that many supportive living or lodge sites use the term "resident". When you see the term "client" used and if you are in supportive living where you use the term "resident", then the word "client" will refer to your "resident".

Section A: A few questions about you

1. Which home care or supportive living site do you work in? (If you work in more than one site; please only list your primary place of work).

____

2. What is your position title? (Check **ONE**; if you have more than one position, check the one that you work most often)

_____ a. Case Manager
_____ b. Registered Nurse
_____ c. Licensed Practical Nurse
_____ d. Health Care Aide/Personal Care Attendant
_____ e. Social Worker
_____ f. Physical Therapist/Assistant
_____ g. Recreational Therapist/Assistant
_____ h. Occupational Therapist/Assistant
_____ i. Pharmacist
_____ j. Dietitian
_____ k. Other (Please specify): _______________________________________

3. How long have you been working in home care or supportive living?

If more than 1 year, how many years? __________ (example: 7 years)
or
If less than 1 year, how many months? __________ (example: 11 months)

4. How long have you been working in this site?

If more than 1 year, how many years? __________ (example: 2 years)
or
If less than 1 year, how many months? __________ (example: 5 months)

Section B: What do you think about the DICE feedback report?

We distributed a report that shows information about clients in your site based on the Resident Assessment Instrument-Home Care (RAI-HC), the tool that is used to collect data about clients in home care and supportive living.

If you work in or cover more than one site, please answer these questions based on the feedback report for the site that you received.

1. Did you receive the report(s)? (Check **ONE** answer**.)**

    _____ a. Yes
    _____ b. No (Obtain a copy(s) to look at to complete this survey)

2. How much of the report did you read? (Check the letter that reflects how much of the report(s) you've read; check only **ONE** answer**.**)

    _____ a. Less than half
    _____ b. About half
    _____ c. More than half
    _____ d. All of it

3. How well do you feel you understood the information that was in the report about clients in your site? (Check the letter that fits how well you understood the information; check **ONE** answer.)

    _____ a. Less than half
    _____ b. About half
    _____ c. More than half
    _____ d. All of it

4. How much do you feel that the information in the report accurately reflects the way your site is? (Check **ONE** answer.)

    _____ a. Does not reflect my site at all
    _____ b. Partially reflects my site
    _____ c. Reflects my site very well

5. How useful did you find the report? (Check **ONE** answer.)

_____ a. Not useful
_____ b. Somewhat useful
_____ c. Useful
_____ d. Very useful

6. Do you think the report showed that your site is doing well or not? (Check **ONE** answer.)

_____ a. The information mostly showed that my site is doing better than the other sites in the study.

_____ b. The information mostly showed that my site is not doing as well as the other sites in the study.

_____ c. The information mostly showed that my site is doing about the same as the other sites in the study.

_____ d. Can't Tell (Please describe): ____

____

____

7. Did you discuss the report with another staff member, either in your site, or someone who works somewhere else in the zone? (Check **ONE** answer.)

_____ a. Yes (***Go to Question 7.1 and 7.2***)
_____ b. No (***Go to Question 8***)

7.1 If **YES** in **Question 7**, what type of staff member did you discuss the report with? (Check **ALL** that apply.)

_____ a. Peer (someone else who does the same type of job as you do)
_____ b. Your direct supervisor
_____ c. A case manager
_____ d. A home care manager
_____ e. Others (Please specify): ____

7.2 If **YES** in **Question 7**, why did you talk to another staff member?
(Check **ALL** that apply.)

_____ i. I wanted to find out what they thought about the report.

_____ ii. I wanted to get their advice about how to make things better for clients based on the report

_____ iii. Other reason (Please tell us what this reason is): ________________________

____

8. Does getting this feedback report make you more interested in other types of data (for example, other domains from the RAI-HC)? (Check **ONE** answer.)

_____ a. Yes (***Go to Question 8.1***)
_____ b. No (***Go to Question 9***)

8.1 **If YES** in **Question 8**, what other kinds of information are you interested in?

____

____

____

9. After reading the report, are there other information that would you like to know from the report? (Check **ONE** answer**.)**

_____ a. Yes (***Go to Question 9.1***)
_____ b. No (***Go to Question 10***)

9.1 If **YES** in **Question 9**, what other information would you like to know from the report that would be MOST helpful to you? (Check **ONE** answer.)

_____ a. Information about the reasons for the information in the report (Why things are the way they are)

_____ b. Information about best practices for specific kinds of care for clients

_____ c. Information about how other sites have addressed similar kinds of concerns

_____ d. Information about whether things are changing over time

_____ e. Other kinds of information (Please describe):

____

____

____

10. Did the report give you information that you could use to make changes in the way you take care of clients? (Check **ONE** answer.)

_____ a. Yes ***(Go to Question 10.1)***
_____ b. No **(*Go to Section C*)**

10.1 **If YES** in **Question 10,** what changes would you like to make in how you take care of clients? (Check **ALL** that apply.)

____ a. Change the way you assess clients

____ b. Change the way you assist clients in their activities of daily living

____ c. Change the daily schedule for clients

____ d. Change policies that affect clients or client care

____ e. I would not change my care practices on my own but the information from the feedback report can be used by other health care providers (e.g a Case Manager, supervisor or others) to make changes to client care

____ f. Other kinds of change (Please describe): ______________________________

____

Section C: Intent to Change Care Practices

Do you provide direct client care?

____ a. Yes (***Answer Questions 1 to 21 and fill out the GENERAL COMMENTS section on the last page***)

____ b. No (***DO NOT answer Questions 1 to 21; Please write your GENERAL COMMENTS in the box provided on the last page***)

All questions refer to changing how you find out if clients you take care of are having pain (Circle ONE number between 1 and 7 for each statement):

1. If I assess or monitor client pain levels, I will feel that I am doing something positive for the client:

   1 2 3 4 5 6 7
   Very unlikely Very likely

2. It causes a lot of worry and concern for a client if I assess or monitor their pain level:

   1 2 3 4 5 6 7
   Very unlikely Very likely

3. If I assess or monitor level of pain, I will detect any problems at an early stage:

   1 2 3 4 5 6 7
   Very unlikely Very likely

4. If I assess or monitor level of pain, I will have to deal with a client more often:

   1 2 3 4 5 6 7
   Very unlikely Very likely

5. The methods I use to assess or monitor level of pain are not very accurate:

   1 2 3 4 5 6 7
   Very unlikely Very likely

6. When I am assessing or monitoring level of pain, I feel rushed:

1 2 3 4 5 6 7
Very unlikely Very likely

7. Having to deal with a client more often is:

1 2 3 4 5 6 7
Extremely undesirable Extremely desirable

8. Doing something positive for the client is:

1 2 3 4 5 6 7
Extremely undesirable Extremely desirable

9. Assessing or monitoring level of pain for each client early and often is:

1 2 3 4 5 6 7
Extremely undesirable Extremely desirable

10. Doing what other caregivers **like me** in my site do is important to me (for example, if you are an RN, think about other RNs in your site):

1 2 3 4 5 6 7
Not at all important Extremely important

11. Doing what experts in home care and supportive living do is important to me:

1 2 3 4 5 6 7
Not at all important Extremely important

12. The approval of the client I take care of is important to me:

1 2 3 4 5 6 7
Not at all important Extremely important

13. People who are important to me think that I should NOT assess or monitor level of pain among the clients I care for:

1 2 3 4 5 6 7
Strongly disagree Strongly agree

14. I expect to assess or monitor level of pain in each client I care for every shift:

1 2 3 4 5 6 7
Strongly disagree Strongly agree

15. I feel under social pressure to assess or monitor clients' level of pain:

1 2 3 4 5 6 7
Strongly disagree Strongly agree

16. I am confident that I can assess or monitor clients' level of pain if I want to:

1 2 3 4 5 6 7
Strongly disagree Strongly agree

17. Whether I assess or monitor clients' level of pain is entirely up to me:

1 2 3 4 5 6 7
Strongly disagree Strongly agree

18. I want to assess or monitor clients' level of pain every shift:

1 2 3 4 5 6 7
Strongly disagree Strongly agree

19. It is expected of me that I assess or monitor clients' level of pain:

1 2 3 4 5 6 7
Strongly disagree Strongly agree

20. I intend to assess or monitor clients' level of pain during each shift:

1 2 3 4 5 6 7
Strongly disagree Strongly agree

21. Out of the next 10 clients you care for, for how many would you expect to assess or monitor level of pain? (Circle **ONE** of the numbers **between 0 and 10** below)

0 1 2 3 4 5 6 7 8 9 10

GENERAL COMMENTS:

This is the end of the survey. Thank you.

Date completed: ____
