# Supplementary material for: Data for improvement and clinical excellence: protocol for an audit with feedback intervention in home care and supportive living
Source: Implement Sci. 2012 Jan 18;7:4. doi: 10.1186/1748-5908-7-4 (PMC3292450; doi:10.1186/1748-5908-7-4)
Supplement: Additional file 2 — Study team. This file contains the project team member descriptions at the time of implementation of DICE Phase II-Home care and Supportive Living. [file 1748-5908-7-4-S2.PDF]

Study Team

Project team member descriptions at the time of implementation of DICE Phase II – Home care and Supportive Living[1]

Kimberly Fraser, RN, PhD, is Research Lead. She is Assistant Professor in the Faculty of Nursing at the University of Alberta. Her research program includes a strong emphasis on decision-making, resource allocation, case management in home care, and related health policy. She will take the lead in designing and conducting the research project, as well as in translating findings both in the practice and policy communities.

Corinne Schalm, MSG, MPA, is Decision-Maker Lead. She is the Vice President of the Shepherd's Care Foundation, Edmonton, Alberta. She is a Certified Health Executive with over 20 years experience in health care planning and administration. She has been a frequent participant on research teams; she was a key participant in the CHSRF-funded Knowledge Brokering Group (KBG) in the Edmonton area. She will participate in the design and conduct of the research project, and take the lead in disseminating findings to the decision-maker and policy communities both in Alberta and nationally.

Marian Anderson, RN, GNC is the Director of Care and Site Lead at Shepherd's Care Millwoods Care Centre. She is a decision-maker, and will assist in conducting the project as well as disseminating to nursing directors in continuing care organizations; she will also contribute to capacity-building by helping to develop training programs for managers and frontline providers.

Wendy Duggleby, RN, PhD, is Professor in the Faculty of Nursing and Endowed Nursing Research Chair in Aging and Quality of Life. Her areas of research interest include Gero-oncology, psychosocial oncology, family caregivers, palliative and end of life care. She is a researcher, and will participate in conducting the research and in disseminating through academic channels as well as through the practice based research network she is developing in aging and quality of life.

Carole Estabrooks, RN, PhD, is Professor in the Faculty of Nursing and Canada Research Chair in Knowledge Translation at the University of Alberta. She is a researcher, and will participate in conducting the research and in disseminating through academic channels as well as through the practice based research network she is developing in continuing care. Her expertise in knowledge transfer and exchange provides a significant asset to the team.

Wendy Harrison, RN, BN, is Executive Director, Seniors Health, North Zone, Alberta Health Services. She is a decision-maker partner, and will participate in conducting the project in home care settings, and will also disseminate findings through the practice and policy communities.

Vivien Lai, MA, is the Senior Policy Advisor in the Strategic Directions Division of Alberta Health and Wellness. She is a decision-maker at a senior policy level, and will provide advice and consultation

[1] Several team members have changed positions since the project was funded. We provide the information current at the time of the second phase of DICE study was implemented

throughout the project and its development, and participate in knowledge transfer and exchange in the practice and policy communities.

Lili Liu, PhD, is Professor and Chair of the Department of Occupational Therapy, Faculty of Rehabilitation Medicine at the University of Alberta. She is a researcher, and will participate in conducting the research and in dissemination, particularly through her strong connection to the rehabilitation health care community.

Suzanne Maisey, MA, is Director of Quality Improvement Projects, Shepherd's Care Foundation, Edmonton, Alberta. She is a decision-maker, and will participate in designing and conducting the project, as well as in knowledge transfer and exchange in the practice community; she was also an active participant in the CHSRF-sponsored KBG.

Lynne Mansell, MHSA, is Executive Director, Clinical Compliance, Seniors Health, Alberta Health Services. She is a decision-maker partner, and will participate in conducting the project in home care settings, and will also disseminate findings through the practice and policy communities.

Colleen Maxwell, PhD, is Associate Professor in the Departments of Community Health Sciences (Centre for Health & Policy Studies) and Medicine (Division of Geriatric Medicine), University of Calgary. She is a researcher, and will participate in disseminating findings both through traditional academic channels and through practice and policy channels; her contacts in other regions in Alberta will be invaluable in knowledge transfer and exchange; she is well-known throughout the province because of an existing project in LTC and assisted living.

Iris Neumann, BSc MSc, is Chief Executive Officer of CapitalCare. She is a decision-maker and will participate in knowledge transfer and exchange in the decision-maker and policy communities.

Brant Poirier is Area manager, Senior Health, Central Zone, Alberta Health Services. He is a decision maker and will participate in designing and conducting the project in Central Zone sites as well as in knowledge transfer and exchange in the practice community.

Barbara Proudfoot, BScN, is Director Integrated RAI Initiatives, RAI-HC, Seniors Health, Alberta Health Services. She provides specialist expertise and leadership in the use of the RAI-HC in Alberta including clinical application and quality initiatives. She is a decision-maker and will participate in designing and conducting the project as well as in knowledge transfer and exchange in the practice community.

Anne Sales, RN, PhD, is the Acting Director for the VA Inpatient Evaluation Centre and is an adjunct faculty member in the Faculty of Nursing at the University of Alberta. Her research program includes a strong emphasis on highly applied research in quality improvement using existing data. She took the lead in designing and conducting DICE in LTC, is a research partner for DICE in HC/SL, and will assist in translating findings both in the practice and policy communities.

Glenda Stein is RAI Lead, Standards Compliance and Accountability, Calgary Zone, Alberta Health Services. She is a decision maker and will participate in designing and conducting the project in Calgary zone sites as well as in knowledge transfer and exchange in the practice community.

Sharon Warren, PhD, is Professor and Director of Rehabilitation Research in the Faculty of Rehabilitation Medicine at the University of Alberta. She is a researcher and will participate in designing and conducting the study, and in disseminating findings through the research community, particularly in rehabilitation health care.
