# Supplementary material for: Data for improvement and clinical excellence: protocol for an audit with feedback intervention in home care and supportive living
Source: Implement Sci. 2012 Jan 18;7:4. doi: 10.1186/1748-5908-7-4 (PMC3292450; doi:10.1186/1748-5908-7-4)
Supplement: Additional file 3 — Feedback report. This file contains an example of the feedback report used in Home care/Supportive living sites. [file 1748-5908-7-4-S3.PDF]

UNIVERSITY OF ALBERTA

Data for Improvement and Clinical Excellence (DICE)

- We have developed feedback reports from the Resident Assessment Instrument-Home Care (RAI-HC), to help care providers in home care and supportive living improve the quality of care for clients.
- The RAI-HC data was used to prepare these feedback reports. The data that you see in this report compares your client population to client populations in other home care and lodge sites in Alberta participating in this study.
- We will hand out the feedback reports every quarter for 4 quarters beginning in June, 2011. The RAI-HC data you see in this report was taken from assessments that were completed between April, 2010 and March, 2011. This one-year data was divided into 4 quarters. Each quarter then shows only data of clients with assessments done in that particular quarter.
- The graphs are in 2 colors – green represents all other home care and lodge sites and the other color is your site.
- Each point in the line shows a percentage. The percent is the number of clients with the health condition (for example, pain) for a particular quarter divided by the total number of clients who have had an assessment for that quarter. A proportion is calculated each quarter for your site and one for the other home care and lodge sites.
- The figures at the right side of the graph show the number of clients included each quarter.
- We would like you to read the feedback report and think about how it represents your site. If you have time and are willing, we also would like you to participate in the survey that asks questions about the feedback report. We want you to do the survey after you have reviewed the feedback report. The survey takes about 10 minutes to complete. If you missed the information session on the study, you can read the information sheet provided in the package. Please put the completed survey in the stamped and addressed envelope provided and mail it back to us.
- If you have any questions about the survey, you can contact:
    - Gloria Gao (780-492-2956), the project coordinator, or
    - Dr. Kimberly Fraser (780-492-7283) who is the researcher conducting this study

DICE Project
Feedback Report, June 2011

➢ PAIN

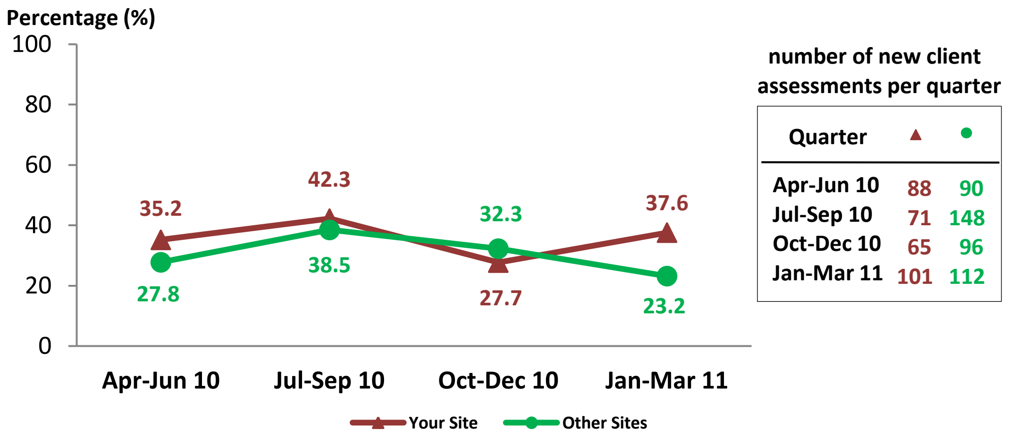

Figure 1. Clients with moderate to severe pain

- The information in Figure 1 is from items k4a and k4b of the RAI-HC
- These two items are used to measure the proportion of clients with moderate to severe pain
- In the January-March 2011 quarter, there was a small increase in the proportion of clients in your site with moderate to severe pain; it was higher than the other sites

➢ FALLS

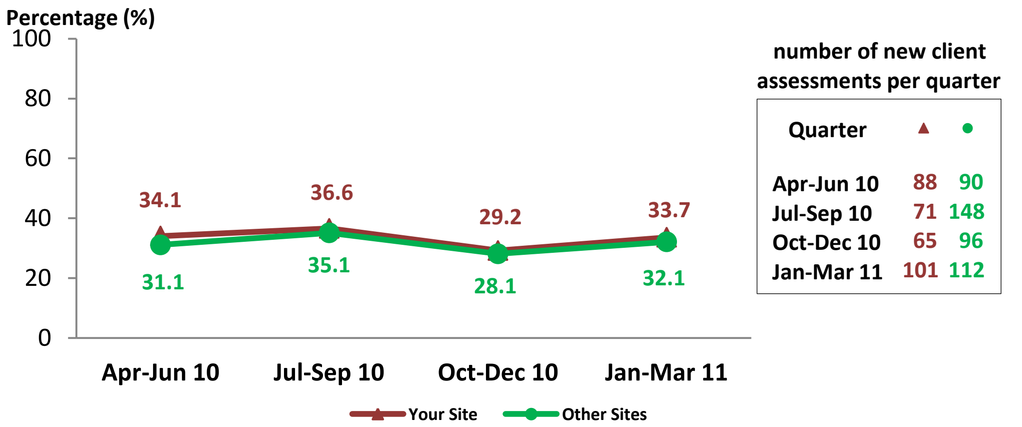

Figure 2. Clients who experienced a fall in the past 90 days

- The information in Figure 2 is from item k5 of the RAI-HC
- This item is used to measure the proportion of clients who experienced a fall in the past 90 days
- In the January-March 2011 quarter, there was almost no change in the proportion of clients in your site who experienced a fall in the past 90 days; it was about the same as the other sites

DICE Project
Feedback Report, June 2011

➢ DELIRIUM

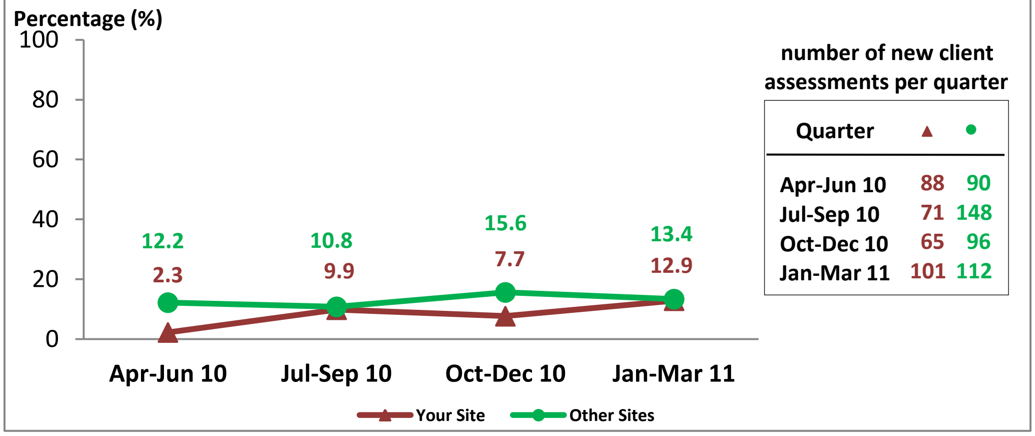

Figure 3. Clients with indications of delirium

- The information in Figure 3 is from items b3a and b3b of the RAI-HC
- These two items are used to measure the proportion of clients with indications of delirium
- In the January-March 2011 quarter, there was almost no change in the proportion of clients in your site who had indications of delirium; it was about the same as the other sites

➢ HOSPITALIZATION

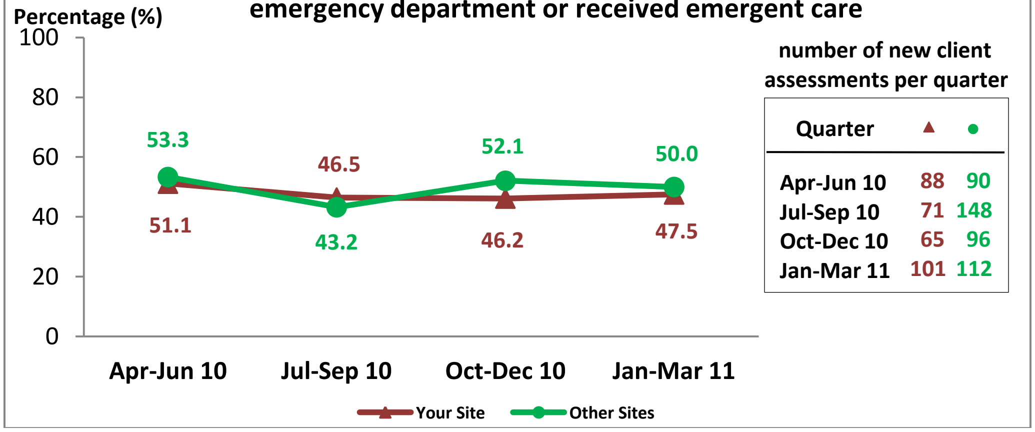

Figure 4. Clients who have been hospitalized, visited hospital emergency department or received emergent care

- The information in Figure 4 is from items p4a, p4b and p4c of the RAI-HC
- These three items are used to measure the proportion of clients who have been hospitalized, visited hospital emergency department or received emergent care
- In the January-March 2011 quarter, there was almost no change in the proportion of clients in your site who have been hospitalized, visited hospital emergency department or received emergent care; it was about the same as the other sites
